# Uncertainty Due to Finite Resolution Measurements

**DOI:** 10.6028/jres.113.011

**Published:** 2008-06-01

**Authors:** S. D. Phillips, B. Toman, W. T. Estler

**Affiliations:** National Institute of Standards and Technology, Gaithersburg, MD 20899

**Keywords:** digitization, ISO 14253-2, measurement, resolution, Sheppard’s correction, standard deviation, uncertainty

## Abstract

We investigate the influence of finite resolution on measurement uncertainty from the perspective of the *Guide to the Expression of Uncertainty in Measurement* (GUM). Finite resolution in a measurement that is perturbed by Gaussian noise yields a distribution of results that strongly depends on the location of the true value relative to the resolution increment. We show that there is no simple expression relating the standard deviation of the distribution of measurement results to the associated uncertainty at a specified level of confidence. There is, however, an analytic relation between the mean value and the standard deviation of the measurement distribution. We further investigate the conflict between the GUM and ISO 14253-2 regarding the method of evaluating the standard uncertainty due to finite resolution and show that, on average, the GUM method is superior, but still approximate.

## 1. Introduction

The issue of measurement uncertainty is becoming increasingly relevant to both calibration laboratories and factory floor metrology. In some cases measurement uncertainty has significant economic impact on the cost of a product. For example, the ISO standard 14253-1 [1] (default condition) requires the expanded uncertainty to be subtracted from both ends of the product tolerance yielding a smaller “conformance zone.” A measurement result must lie in this zone in order for the manufacturer to distribute the product. Avoiding overestimation of the measurement uncertainty can result in a larger conformance zone and hence lower product cost. In some situations the finite resolution of a measuring instrument is a significant contributor to the uncertainty statement. For example, the manufacturers of hand held digital calipers typically set the resolution of the instrument such that repeated measurements of the same quantity yield nearly the same result, within one or two units of the least count (i.e., resolution) of the instrument. In principle the caliper would be more accurate with an additional display digit, however, customers often perceive quality as the ability of the instrument to yield the same value for repeated measurements. The uncertainty budget of a measurement performed with a hand held instrument typically contains only a few significant influence quantities since most quantities such as the accuracy of its calibration (typically reported at 20 °C) and thermal effects associated with the measurement are usually small compared to the instrument resolution. Therefore the inclusion (or not) of the uncertainty associated with the finite resolution of the display can significantly affect the magnitude of the reported uncertainty. Similar issues may arise with the finite resolution introduced by analog to digital conversion electronics where repeated sampling of a signal differs by only one or two bits.

In this paper we first examine some general properties of the probability distribution associated with a measurement recorded with finite resolution and perturbed with Gaussian noise. The distribution of the measurement results is generated via computer simulation so that we can control the underlying population mean (true value) and standard deviation (of Gaussian noise) and produce large quantities of recorded measurement results. We then examine how the finite resolution and measurement noise impacts the uncertainty evaluation under different measurement scenarios.

The *Guide to the Expression of Uncertainty in Measurement* (GUM) [2] identifies the finite resolution of a measuring instrument as a contributor to measurement uncertainty of a measurement from that instrument. It further suggests that this effect should be evaluated by a (Type B) uniform distribution with a full width of one resolution unit, resulting in a standard uncertainty of $\frac{1}{\sqrt{12}}$ in units of the resolution (GUM F.2.2.1). The GUM also suggests that this contribution is uncorrelated to other uncertainty sources and that it should be added in a root-sum-of-squares (RSS) manner in the uncertainty statement (e.g., see GUM H.6).

Alternatively, ISO 14253-2 [3] recommends examining the standard uncertainty of the resolution relative to the standard deviation of repeated measurements and then including the larger of the two in the uncertainty evaluation and discarding the lesser of the two. The rationale of this method is that the resolution is already intertwined with the standard deviation of repeated measurements, since that data is recorded with the resolution of the instrument.

We summarize these two procedures as:

Rule 1: RSS the standard uncertainty of the resolution (Type B via a uniform distribution) with the uncertainty associated with the standard deviation of repeated measurements in the uncertainty evaluation.
Rule 2: Include the larger of the following two: the standard uncertainty of the resolution (Type B via a uniform distribution) and the standard deviation of repeated measurements in the uncertainty evaluation and discard the smaller of the two.

Clearly Rule 1 and Rule 2 cannot both simultaneously be the best estimate of the uncertainty for all measurement cases.

There are two measurement scenarios we will consider. The “special test” scenario involves constructing an uncertainty statement for one specific measurement quantity. Typically this will involve repeated observations of the quantity, each recorded with finite resolution. The best estimate of the measurand is considered to be the mean of the repeated observations (after all corrections are applied) and the uncertainty statement will be associated with this mean value.

The “measurement process” scenario involves constructing an uncertainty statement that will be applicable to a series of future measurement results. This is typical of commercial metrology where a large number of nearly identical artifacts or workpieces are produced and only a single measurement, having finite resolution, is recorded. In this scenario, the uncertainty statement is developed once and then associated with each future (single observation) measurement result. The uncertainty evaluation process will involve, among other things, repeated measurements of calibrated artifacts, recorded with finite resolution, and used to characterize the measurement variation.

Without loss of generality we consider the case where a resolution increment has the value of unity, and the infinite resolution value of the measurement, *μ*, lies between zero and one-half (all other cases are modulo this problem). We consider the case where the measurement is corrupted by noise from a Gaussian distribution with standard deviation *σ*. The situation of interest will be for *σ* < 1 since large *σ* is equivalent to infinite resolution. Our approach will be to examine both the special test scenario and the measurement process scenario in the limit of large sample size.

## 2. Probability Distributions

**Fig. 1:**
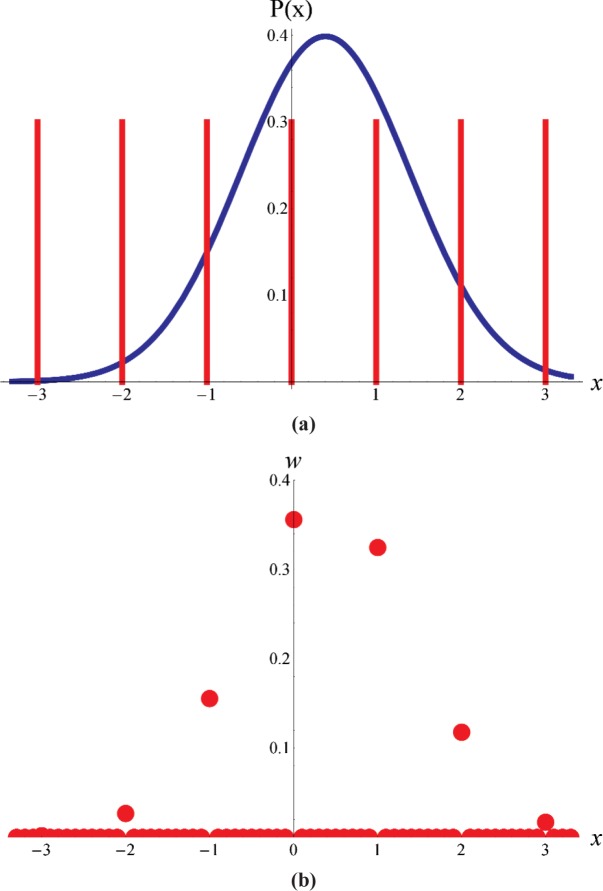
(a) A Gaussian distribution with *μ* = 0.4 and *σ* = 1 together with a comb function representing the instrument’s finite resolution. (b) The corresponding discrete distribution with values at the integer values equal to the probability mass function.

In the limit of large sample size, expectation values are estimated by sample statistics. Finite resolution requires that only integer values will be observed. Hence a continuous probability distribution function (pdf) becomes a discrete pdf that can be written:

$$\begin{array}{l} \text{Discrete}\;\text{pdf}\;(x)=w(\mu ,\sigma ,n)\;\delta (x-n)\\ \;n:-\infty ,\ldots ,-3,-2,-1,0,1,2,3,\ldots ,\;+\infty \\ \;\text{with}\;w(\mu ,\sigma ,n)=\int_{n-\frac{1}{2}}^{n+\frac{1}{2}}\text{pdf}\;(\mu ,\sigma ,x)\;dx\\ \;=\Phi \left(\mu ,\sigma ,n+\frac{1}{2}\right)-\Phi \left(\mu ,\sigma ,n-\frac{1}{2}\right) \end{array}$$ (1)

where *w* is the probability mass function, *δ* (*x* − *n*) is a delta function, and Φ is the cumulative probability distribution function. Figure 1 illustrates the quantization (using Eq. (1)) of a Gaussian distribution with *μ* = 0.4 and *σ* = 1.

## 3. Descriptive Statistics: Mean and Standard Deviation

Using the discrete probability distribution, expectation values can be calculated. The expected value of the mean, $\bar{x}$, and the standard deviation, *s*, can be calculated from:

$$\begin{array}{l} \bar{x}(\mu ,\sigma )=\sum_{n=-\infty }^{+\infty }w(\mu ,\sigma ,n)\;n\;\text{and}\\ s(\mu ,\sigma )=\sqrt{\sum_{n=-\infty }^{+\infty }w(\mu ,\sigma ,n)\;{\left(n-\bar{x}\right)}^{2}}. \end{array}$$ (2)

**Fig. 2:**
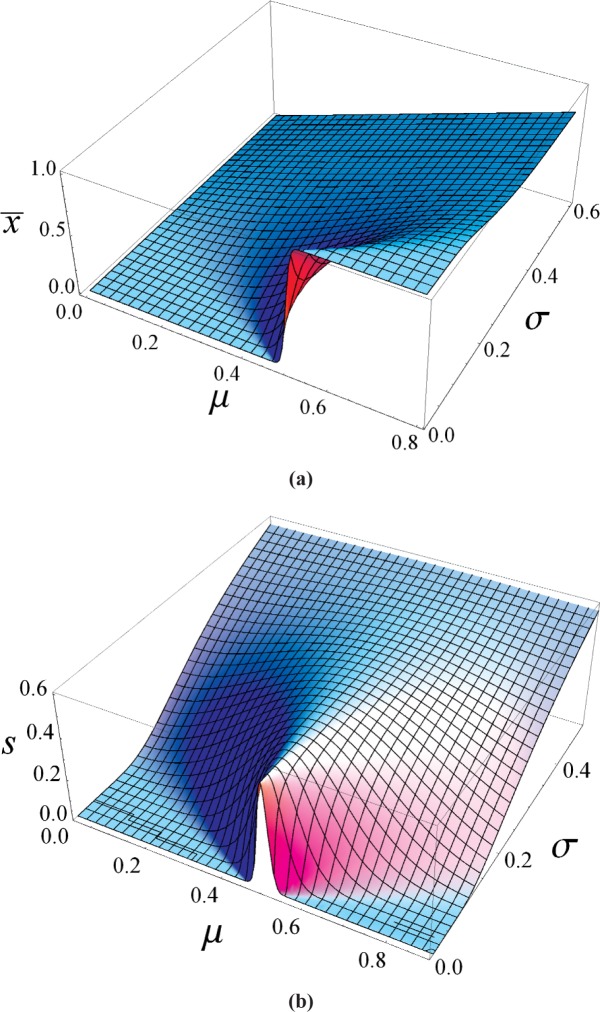
Plots of (a) the sample mean, $\bar{x}$, and (b) sample standard deviation, *s*, as a function of *μ* and *σ* for data recorded with a resolution of unity.

Figure 2 displays plots of $\bar{x}$ and *s* for relevant values of *μ* and *σ*. The plot for $\bar{x}$ shows the expected step function (due to the discrete resolution) at *μ* = 0.5 when *σ* → 0, and the expected behavior $\bar{x}\to \mu$ for large *σ*. Similarly, the plot for *s* displays the expected rapid rise when *μ* → 0.5 and *σ* is small, and the expected behavior *s* → *σ* for large *σ*.

**Fig. 3:**
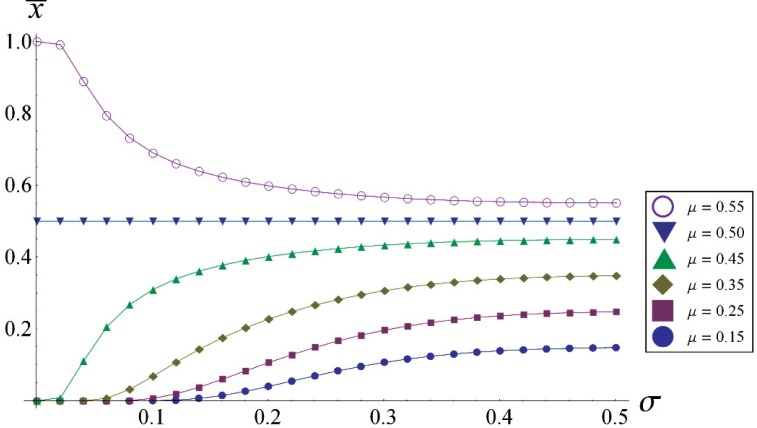
Plot of $\bar{x}$ vs. *σ* for various values of *μ*; note that for any particular $\bar{x}$ the possible values of *μ* are always greater than $\bar{x}$ resulting in a bias of $\bar{x}$ toward the resolution unit.

An interesting and intuitive feature of Fig. 2 can be more easily seen in Fig. 3, which shows the mean $\bar{x}$ as a function of *σ* for various values of *μ*. For any given value of $\bar{x}\leq 0.5$, which is a horizontal line on Fig. 3, the possible values of *μ* are always greater than $\bar{x}$. (Equivalently stated, for 0 ≤ *μ* ≤ 0.5 and any *σ*, then $\bar{x}\leq \mu$). Hence the observed discrete population mean is always biased toward the closest resolution increment regardless of the value of *σ* (for large *σ* this bias becomes insignificant). For small *σ*, e.g., *σ* < 0.5, it is incorrect to believe that uncertainty associated with the sample mean approaches zero as a result of averaging a large sample; rather it approaches a fixed value resulting in a systematic error.

**Fig. 4:**
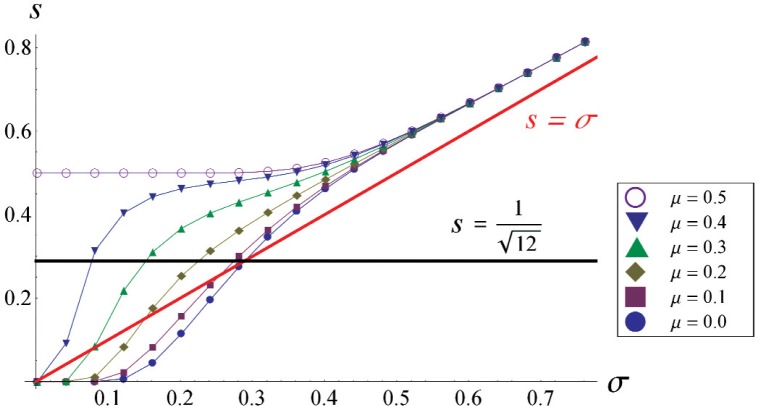
Plot of the standard deviation of the finite resolution population as a function of the standard deviation of the Gaussian noise.

Figure 4 shows the dependence of *s* on *σ* for various values of *μ*. As *σ* becomes large, *s* → *σ*, approaching it from above. The slight overestimation of *σ* by *s* is well known and the difference is called “Sheppard’s correction” [4]. We further point out that *s* ≥ *σ* when $s>\frac{1}{\sqrt{12}}$ regardless of *μ*. Similarly, $\frac{1}{\sqrt{12}}\geq \sigma$ when $s<\frac{1}{\sqrt{12}}$ regardless of *μ*; hence Max $\left[\frac{1}{\sqrt{12}},s\right]$ ≥ *σ* for all *μ*, a fact we will make use of later.

**Fig. 5:**
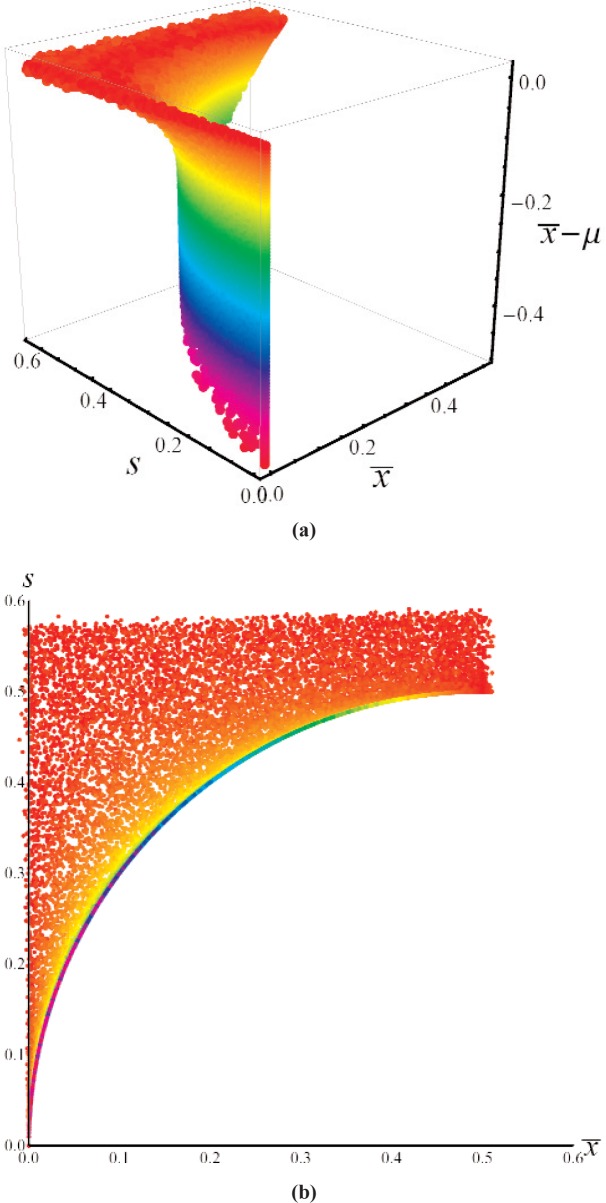
(a) the value of $\bar{x}$, *s*, and $\bar{x}-\mu$ for all combinations of 0 ≤ *μ* ≤ 0.5 and 0 ≤ *σ* ≤ 0.5; (b) the 2D plot of the $\bar{x}$, *s*, coordinates shown in (a) using the same color coding for the systematic error.

What is not obvious from examining the previous plots is the interaction of $\bar{x}$ and *s*. In particular, there are combinations of $\bar{x}$ and *s* that are forbidden. We examine this by creating a dense grid of (*μ*, *σ*) pairs over the domain 0 ≤ *μ* ≤ 0.5 and 0 ≤ *σ* ≤ 0.5. For each (*μ*, *σ*) we create (numerically, with a sample size of 10,000) a finite resolution sample of Gaussian distributed values and calculate the corresponding mean $\left(\bar{x}\right)$ and standard deviation (*s*). Figure 5 shows a plot of the resulting $\bar{x}$, *s* space, showing that there is a disk with a radius of one-half resolution unit, as described in Eq. (3), inside which no combination of $\bar{x}$ and *s* values can occur.

$$\begin{array}{l} {\left(\frac{1}{2}-\bar{x}\right)}^{2}+s^{2}\\ ={\left(\frac{1}{2}\right)}^{2}+\sum_{n=-\infty }^{n=+\infty }w(n,\mu ,\sigma )\left(n^{2}-n\right)\geq {\left(\frac{1}{2}\right)}^{2}\;\forall \mu ,\sigma . \end{array}$$ (3)

The dense set of $\bar{x}$, *s* coordinates that occur on the disk boundary is understood by considering values of *n* where the equality in Eq. (3) holds, i.e., the disk boundry. Since each term in the infinite sum in Eq. (3) must be positive or zero, the equality can only hold when infinite sum of the terms *w*(*μ*, *σ*, *n*)(*n*^2^ − *n*) is zero. This occurs when the weight functions *w*(*μ*, *σ*, 0) + *w*(*μ*, *σ*, 1) = 1. That is, for any *μ* (0 ≤ *μ* ≤ 0.5) and *σ* such that $\Phi \left(\mu ,\sigma ,1\frac{1}{2}\right)-\Phi \left(\mu ,\sigma ,-\frac{1}{2}\right)=1$ then all of the probability is contained in the weight functions *w*(*μ*, *σ*, 0) and *w*(*μ*, *σ*, 1) and hence the infinite sum of the terms *w*(*μ*, *σ*, *n*)(*n*^2^ − *n*) is zero. Hence, when the equality of Eq. (3) holds the values of $\bar{x}$ and *s* satisfy the equation of a circle with a radius one half resolution unit. (Since all Gaussian distributions have some probability, albeit infinitesimal, to extend to infinity, the disk radius is actually a limiting value.)

The Z dimension in Fig. 5 corresponds to the systematic (i.e., expected) error $\bar{x}-\mu$ that is associated with *σ*) point. As previously described, the expected error is always biased toward the resolution unit, i.e., the bias is negative as shown in the Z coordinate of Fig. 5. Furthermore, as seen in the figure, it is only the values where ${\left(\frac{1}{2}-\bar{x}\right)}^{2}+s^{2}={\left(\frac{1}{2}\right)}^{2}$ that are significantly biased while other values of $\bar{x}$, *s* away from this disk edge rapidly become an unbiased estimator of *μ*.

**Fig. 6:**
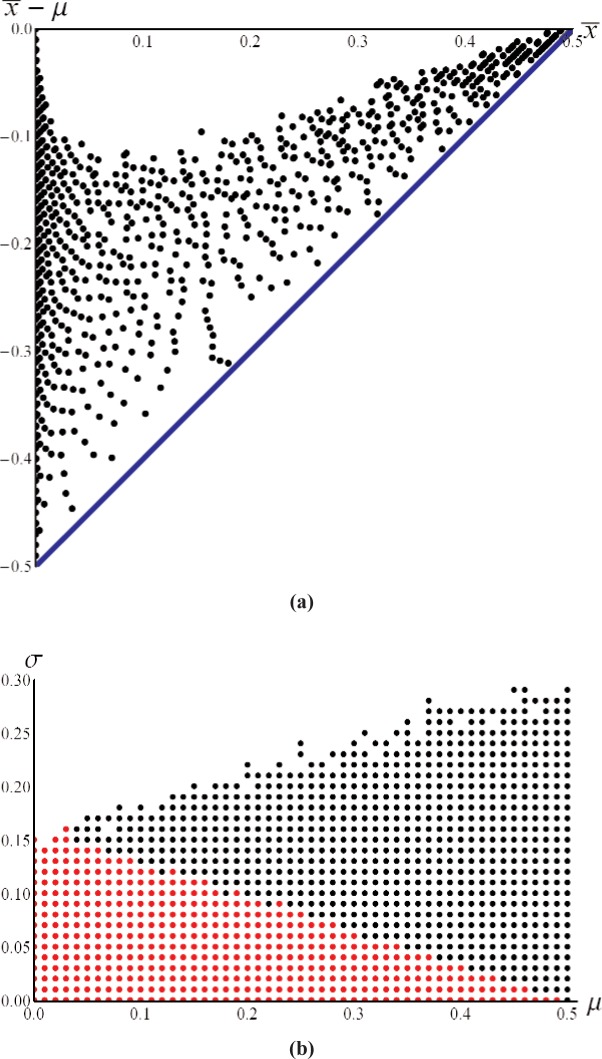
(a) the error $\left(\bar{x}-\mu \right)$ for points on the disk boundary shown in Fig. 5 together with the bounding curve $\bar{x}-½$ shown in blue. (b) The points in *μ*, *σ* space that map to the disk boundary; the red points all map very close to $\bar{x}=0$, *s* = 0.

The magnitude of the systematic error, $\bar{x}-\mu$, values that lie on the disk edge of Fig. 5 is shown in Fig. 6(a). Also shown in Fig. 6(a) is the lower bound of the expected error given by $\bar{x}-½$. Note that the range of errors for a given $\bar{x}$ results from different *μ*, *σ* points mapping to the same value of $\bar{x}$, but having different systematic errors. Figure 6(b) shows the points in *μ*, *σ* space that map to values of $\bar{x}$, *s* that lie on the disk boundary; this represents a significant region of *μ*, *σ* space and thus gives rise to the density of $\bar{x}$, *s* points on the disk boundary shown in Fig. 5. The points shown in red in Fig. 6(b) all map very close to the point $\bar{x}=0$, *s* = 0.

It is tempting to apply a correction for the average systematic error (as a function of $\bar{x}$) to obtain a better estimate of *μ*. However, since the bias is nearly a step function that rapidly approaches zero away from the disk edge, applying such a correction based on the proximity of the $\bar{x}$, *s* coordinate to the disk edge is subject to misapplication due to statistical variations expected in any finite sample size. A simpler but cruder approximation would be to use the uncorrected value of $\bar{x}$ and consider the systematic error to be bounded between zero and the line $\bar{x}-0.5$ whenever *s* ≤ 0.6. We will consider an uncertainty rule based on this approximation for the special test scenario.

## 4. Errors and Level of Confidence

For the discrete pdf given by Eq. (1) with standard deviation given by Eq. (2) we can explore the relationship between the standard deviation and the level of confidence, i.e., the coverage factor. Although we are considering random Gaussian noise corrupting our measurement result, the subsequent rounding due to finite resolution significantly changes the level of confidence for a coverage factor of *k* = 2, particularly when *σ* < 0.5. We can gain some insight into this effect by examining the magnitude of the error at the 95th percentile in relation to the magnitude of the standard deviation. The magnitude of the error that occurs at the 95th percentile is given by the cumulative probability distribution associated with Eq. (1) and evaluated to determine the integer, *n*_95%_, and the corresponding error value, *ε*_95%_, by increasing the number of terms in Eq. (4) until the inequality just holds.

$$\begin{array}{l} w(0,\mu ,\sigma )+w(1,\mu ,\sigma )+w(-1,\mu ,\sigma )+w(2,\mu ,\sigma )\\ \;+\ldots +w(n_{95\%},\mu ,\sigma )\geq 0.95\;\text{and}\;\varepsilon_{95\%}=\left|n_{95\%}-\mu \right|. \end{array}$$ (4)

**Fig. 7:**
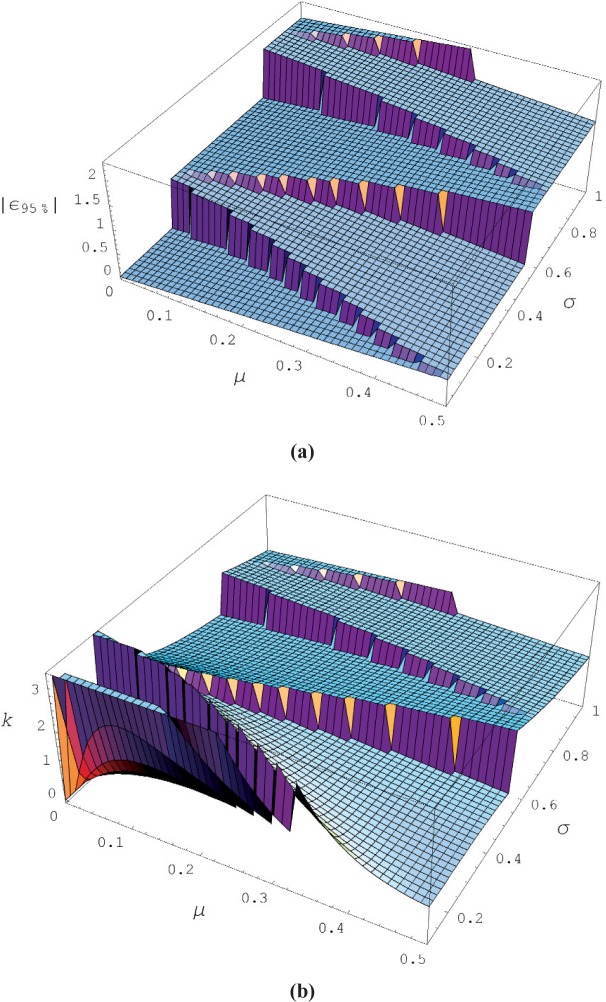
(a) The 95th percentile error (in units of resolution) as a function of the true value (*μ*) and the standard deviation of the Gaussian noise (*σ*); (b) the corresponding coverage factor required to include the 95th percentile error.

Clearly *ε*_95%_ will depend on the particular values of *μ* and *σ*, and we expect that *ε*_95%_ will also display the discreteness of the underlying discrete pdf. For example, as *σ* increases with *μ* fixed, *ε*_95%_ will remain fixed until the inequality in Eq. (4) is violated forcing the addition of another term to the cumulative probability and a corresponding jump in the value of *ε*_95%_. Figure 7(a) shows the *ε*_95%_ value over the region: 0 ≤ *μ* ≤ 0.5 and 0 ≤ *σ* ≤ 1. Figure 7(b) displays the required coverage factor to achieve a 95 % level of confidence, i.e., *k* = *ε*_95%_/*s*. For *μ* small but nonzero and *σ* small, the coverage factor approaches infinity since all the measured values of *x* yield zero, resulting in *s* → 0 while *x* − *μ* remains finite. One method to avoid infinite coverage factors is to establish a finite lower bound for the standard uncertainty; this is employed by Rules 1 and 2.

## 5. Measurement Process Scenario

In the measurement process scenario an uncertainty evaluation is performed on a measurement system and then a future (single observation) measurement result, *x*, which represents the best estimate of the measurand is assigned an uncertainty based on the prior evaluation. Since *x* is a single reading from an instrument having resolution *R*, and in this paper we take *R* = 1, hence *x* must be an integer.

One complicating factor in this scenario is that the reproducibility evaluation is conducted on a reference artifact having some value *μ*_Ref_ while the uncertainty statement is relevant to a future measurement having a different (infinite resolution) value *μ*, and the two values are independent. For example, consider calibrating 100 mm long artifacts with an instrument having 0.01 mm of resolution. The reference artifact on which the uncertainty evaluation is based might be 100.002 mm long, where as the artifacts to be measured in the future will each have some other value, e.g., 99.997 mm. Hence all of the measurements used in the uncertainty evaluation to determine *s* (based on a large sample of measurements) will depend on the particular value of *μ*_Ref_, and different values of *μ*_Ref_ will yield different values of *s*; see Fig. 2b. Note that the best estimate of the reference artifact, ${\bar{x}}_{\text{Ref}}$, is discarded since it provides no information about the value of the future measurement result.

In this paper we will examine only two values for *μ*_Ref_. It is frequently the case that reference artifacts used in uncertainty evaluations have a value that is exactly on a unit of resolution; we denote this case as *μ*_Ref_ = 0. An example of this would be a digital caliper reading in units of inches that is evaluated by examining repeated measurements on gauge blocks from an English (inch based) set. The caliper may have a resolution of 0.001 inch and the gauge blocks will be within a few microinches of some multiple of 0.001 inch. (Note for this condition to hold, the systematic error of the instrument must be small with respect to the resolution.)

Alternatively, we examine the case where the reference artifact has a uniform probability of being distributed between 0 and 0.5, we denoted case as *μ*_Ref_ = Σ*μ*. In the previous example of the caliper, this could occur when repeated measurements are made on several different metric gauge blocks using an inch based caliper, and the results are pooled to obtain the standard deviation.

We consider three rules to evaluate the standard uncertainty due to finite resolution and repeatability in the measurement process scenario given in Eq. (5);

$$\begin{array}{l} \text{Rule}\;1:\;u(x)=\sqrt{{\left(\frac{\frac{1}{2}R}{\sqrt{3}}\right)}^{2}+s{\left(\mu_{\text{Ref}}\right)}^{2}}\\ \text{Rule}\;2:\;u(x)=\text{Max}\left[\frac{\frac{1}{2}R}{\sqrt{3}},s(\mu_{\text{Ref}})\right]\\ \text{Rule}\;3:\;u(x)=\sqrt{{\left(\frac{\frac{1}{2}R}{\sqrt{3}}\right)}^{2}+{\left(\text{Max}\left[\frac{\frac{1}{2}R}{\sqrt{3}},s(\mu_{\text{Ref}})\right]\right)}^{2}} \end{array}$$ (5)

where *R* is the resolution (in this paper *R* = 1), and *s*(*μ*_Ref_) is the standard deviation of a large sample of measurements on an artifact with a (infinite resolution) value *μ*_Ref_.

Rules 1 and 2 are based on the GUM and ISO 14253-2 respectively, as previously described. Rule 3 includes the observation that $\text{Max}[\frac{1}{\sqrt{12}},s]\geq \sigma$.

All three rules contain two quantities, one dependent on *R* and the other on *s*, and a prescription to combine them. The first quantity (with *R* = 1) has the value $\frac{1}{\sqrt{12}}$ and represents the standard uncertainty associated with a uniform distribution with limits ± ½, representing the fact that the single (future) observation *x* could be located anywhere in the resolution interval. The second quantity is an estimate of the underlying population variance *σ*, which gives rise to the observed variation in *x*. That is, the first quantity accounts for the ambiguity due to the resolution and the second quantity accounts for the reproducibility of the measurement result.

Rule 1 always uses the calculated standard deviation as an estimate for *σ*. However, when *σ* is small, *s* can underestimate *σ*, e.g., if *μ* = 0 and *σ* = 0.2 nearly all of the repeated observations will be zero and hence *s* ≈ 0. Rule 2 also estimates *σ* using *s* and then selects the larger of *s* or the standard uncertainty of the resolution. We can expect that by omitting one of the two uncertainty sources, that Rule 2 will underestimate the uncertainty associated with the measurand. We introduce Rule 3 that estimates *σ* by selecting a quantity that is an upper bound for *σ*, as previously noted in Sec. 3.

## 6. Testing the Uncertainty Rules for the Measurement Process Scenario

We can estimate how effective these rules are in producing expanded uncertainty intervals that contain the reasonable values that can be attributed to the measurand. In the measurement process scenario, the single measurement result, *x*, is determined by the value of *μ* (which is a property of the object but is unknown), corrupted by a random perturbation arising from *σ*, and rounded to the resolution unit. We can imagine that this measurement is repeated a large number of times and then ask what fraction of the errors are contained in the uncertainty statement, where the error is *ε* = *x* − *μ*, and is due to the random Gaussian perturbation and the rounding to an integer value. We will adopt this viewpoint since, in commercial metrology, it is typical to construct an expanded uncertainty using the coverage factor *k* = 2 and then expect that 95 % of the potential measurement errors will be contained within this interval.

One complicating factor in the measurement process scenario is that the probability that a future measurement result will be contained within the expanded (*k* = 2) uncertainty interval is a function of three population variables: *μ*_Ref_, *μ*, and *σ*. Our approach will be to fix the population variables, evaluate the expanded uncertainty, *U*, and then draw a large number of samples (each representing a future measurement) and determine what fraction are contained within the expanded uncertainty interval. We will then select another set of population variables and continue this methodology until we have examined the set of population variables of interest.

**Fig. 8:**
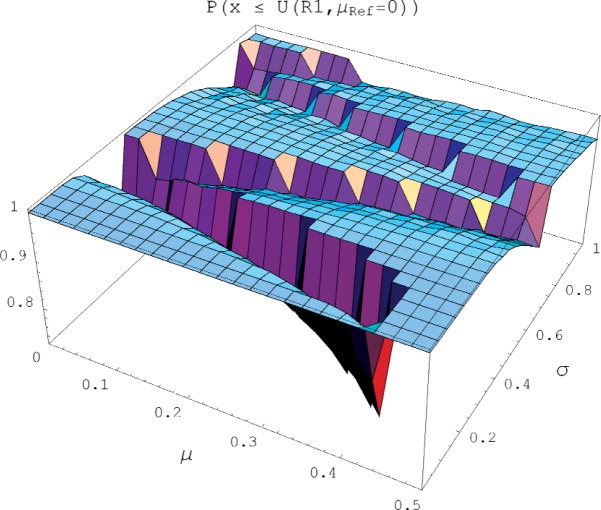
A plot of the probability that the measurement error of a single observation (having value *μ* and corrupted by Gaussian noise of standard deviation *σ*) will be contained within the expanded uncertainty of Rule 1 evaluated with *μ*_Ref_ = 0.

Figure 8 displays the probability that a future measurement result will be contained within the expanded uncertainty interval evaluated by Rule 1 (the GUM rule) with *μ*_Ref_ = 0 over the domain 0 ≤ *μ* ≤ 0.5 and 0 < *σ* ≤ 1. As seen in the figure, the surface consists of a relatively flat mesa having a containment probability near unity, interrupted by abrupt canyons of significantly lower containment probability. The corresponding plots using Rule 2 and Rule 3 are similar, but with broader and deeper canyons, and narrower and shallower canyons, respectively. We note that in Fig. 8, and in the associated three statistics in Table 1, the containment probability only addresses the question of what fraction of errors (for a particular *μ*, *σ*) are contained in the uncertainty interval; it does not address their relative position within the interval. Hence the same containment probability would be assigned to two different distributions having the same fraction of uncontained errors, despite the fact that one distribution might have the uncontained errors only slightly away from the uncertainty interval and the other may have its uncontained errors grossly away from the uncertainty interval.

Table 1 summarizes these results. Min P is the minimum containment probability over the domain of population variables examined. That is, over the domain of *μ*, *σ* values considered, find the particular value of *μ*, *σ* that has the smallest containment probability and report that value as Min P. For example, when Rule 1 is evaluated with *μ*_Ref_ = 0, the minimum containment probability occurs at *σ* = 0.15 and *μ* = 0.4; that is, for this set of values a (randomly chosen) future measurement result has only a 0.75 probability of being contained in the expanded uncertainty interval of this rule. The Min P value tells us something about the lowest point on the surface shown in Fig. 8.

Avg P is the average probability of a measurement error containment over all values of *μ*, *σ* considered; for the previous example of Rule 1 with *μ*_Ref_ = 0, Avg P(*x* ≤ *U*) = 0.97, hence the probability of error containment for most values of *μ* and *σ* is near unity.

Most metrologists expect approximately 95 % of the potential measurement errors to be contained in the expanded (*k* = 2) uncertainty interval. However, even for the infinite resolution case of a Gaussian population, 95 % is the expected fraction of error containment, and any finite sample size will have slightly more, or slightly less, than 95 % of the deviations from the mean contained within 2 *s*. As another measure of the effectiveness of an uncertainty rule, we introduce the percentage of the surface of Fig. 8 that does not include at least a 94 % containment probability; this is denoted % P ≤ 0.94. The value of 0.94 was selected since, for the selected sample sizes, the corresponding infinite resolution case always included at least this fraction of the errors. Note both the % P ≤ 0.94 and Avg P(*x* ≤ *U*) depend on the extent of the *μ*, *σ* domain under consideration, hence they are relative metrics that are only useful when comparing rules over the same *μ*, *σ* domain. (All values in Table 1 and Table 2 were generated numerically, and used sample sizes of 10,000; the uncertainty in the results is estimated at 0.03 resolution units.)

From Table 1 we see that none of the three (ad hoc) rules guarantees 95 % error containment for all values of *μ* and *σ*, with Rule 2 failing this criteria 30 % of the time for the *μ*_Ref_ = 0 case. All of the three rules benefited from computing the standard deviation of the measurement process using many reference standards with different values and pooling the results, i.e., the *μ*_Ref_ = Σ*μ* case. Indeed, for the *μ*_Ref_ = Σ*μ* case, Rule 1 (GUM) performance approached that of Rule 3 without the need to compare the relative size of *s* to the standard uncertainty of the resolution, as required in both Rule 2 and Rule 3.

Also shown in Table 1 are three statistics that describe the difference between the expanded uncertainty and the magnitude of the 95th percentile error, |*ε*_95%_|. This tells us something about the amount of the over (or under) estimation of the uncertainty interval. As might be expected for simple ad-hoc uncertainty rules, none of the three rules adapts itself well to the discontinuous nature of the discrete pdf, e.g., see Fig. 7, and at some values of *μ*, *σ* they significantly overestimate the magnitude of |*ε*_95%_|, while at other points they underestimate its magnitude. These statistics will be of more value for the special test measurement scenario.

## 7. Special Test Scenario

In the special test scenario, repeated observations are used to calculate both the sample mean (which in general will not be an integer) and the standard deviation, and these can be expected to approach the discrete population mean and standard deviation $\bar{x}$, *s*, in the limit of large sample size. Hence, in this scenario the number of observations used to calculate $\bar{x}$, $N_{\bar{x}}$, is the same as the number of observations used to calculate the standard deviation *s*. In the special test scenario, see Eq. (7), the GUM rule (Rule 1) combines the uncertainty of the resolution unit with the standard deviation of the sample mean. For large samples, the uncertainty associated with the resolution unit will clearly dominate since the standard deviation of the mean scales as $1/\sqrt{N_{\bar{x}}}$. Similarly, the ISO rule (Rule 2) selects the larger of the two terms; hence we expect these two rules to be similar since the uncertainty of the resolution unit dominates the standard deviation of the mean in both cases. Consequently, it is clear that for large sample sizes both of these rules overestimate the uncertainty associated with $\bar{x}$.

For Rule 3 we use our observation that near the forbidden disk region (rather crudely described by the region *s* ≤ 0.6) $\bar{x}$ is a biased estimator of *μ* and the value of *μ* is contained in the interval $\bar{x}\leq \mu \leq 0.5$ as seen in Fig. 3. While in principle a correction should be applied to account for this bias, in practice the typical user is unlikely to accommodate such an inconvenience. Consequently, we describe the ignorance in the location of *μ* by a uniform distribution as seen in Rule 3 of Eq. (7). Furthermore, Rule 3 includes the observation that $\text{Max}[\frac{1}{\sqrt{12}},s]\geq \sigma$; outside the disk region Rule 3 treats the uncertainty evaluation as the infinite resolution case.

$$\begin{array}{l} \text{Rule}\;1:\;u(\bar{x})=\sqrt{{\left(\frac{\frac{1}{2}R}{\sqrt{3}}\right)}^{2}+\frac{{\left(s\right)}^{2}}{N_{\bar{x}}}}\\ \text{Rule}\;2:\;u(\bar{x})=\text{Max}\left[\frac{\frac{1}{2}R}{\sqrt{3}},\frac{s}{\sqrt{N_{\bar{x}}}}\right]\\ \text{Rule}\;3:\;u(\bar{x})=\sqrt{{\left(\frac{\frac{1}{2}R-\bar{x}}{\sqrt{3}}\right)}^{2}+\frac{{\left(\text{Max}\left[\frac{\frac{1}{2}R}{\sqrt{3}},s\right]\right)}^{2}}{N_{\bar{x}}}}\text{if}\;s\;\leq 0.6\;R\\ \;=\frac{s}{\sqrt{N_{\bar{x}}}}\;\text{if}\;s\;>0.6\;R \end{array}$$ (7)

## 8. Testing the Uncertainty Rules for the Special Test Scenario

In the case of a large sample size the values of both $\bar{x}$ and *s* become relatively stable and consequently the magnitude of the systematic error $\left|\bar{x}-\mu \right|$ and the uncertainty rule become stable. Hence we can consider the quantity $U-\left|\bar{x}-\mu \right|$ over a region of *μ*, *σ* values to determine whether or not a particular uncertainty rule contains the systematic error.

Table 2 displays the information regarding the containment probability for the three rules as applied to the special test scenario. Both Rule 1 and Rule 2 greatly overestimate the uncertainty associated with the mean and hence always contain 100 % of the errors. Rule 3 more closely approaches the 95 % error containment interpretation with a minimum containment probability of 0.94 and an average (over the *μ*, *σ* values considered) of 0.98.

To consider the magnitude of the overestimation we examine the expected values of the difference between the expanded uncertainty and |*ε*_95%_|. As previously suggested, Rule 1 and Rule 2 behave similarly, and both typically overestimate |*ε*_95%_| by one-half a resolution unit. Rule 3 does significantly better by more closely matching the expanded uncertainty to the |*ε*_95%_| value, with an average overestimation of 0.15 resolution units.

In the special test scenario with a large sample size, Rule 1 and Rule 2 become identical with $\mu (\bar{x})\to \frac{1}{\sqrt{12}}$, and Rule 3 yields $\mu (\bar{x})\to \frac{\frac{1}{2}-\bar{x}}{\sqrt{3}}$ if *s* ≥ 0.6 and $u(\bar{x})\to 0$ if *s* ≥ 0.6. Elster [5] has examined the general solution for the special test scenario using Bayesian calculations. He also considered the special case of data with an equal number of ones and zeros (hence $\bar{x}=0.5$ and *s* = 0.25), and showed for large samples that the best estimate of *μ* was the mean $\bar{x}=½$ and $u(\bar{x})\to 0$; this result is given by Rule 3 but not by Rule 1 and Rule 2, which yield $\frac{1}{\sqrt{12}}$.

## 9. Conclusions

We have examined the statistics associated with data recorded with finite resolution in the limit of large sample size. Due to finite resolution, there exists a disk defined by ${\left(\frac{1}{2}-\bar{x}\right)}^{2}+s^{2}={\left(\frac{1}{2}\right)}^{2}$ inside which the values of $\bar{x}$ are forbidden. Furthermore, values of $\bar{x}$ that occur on the boundary of the disk are always (except for the *μ* = 0 and *μ* = 0.5 case) systematically biased toward the resolution unit. Away from the disk boundary $\bar{x}$ rapidly becomes an unbiased estimator of *μ*.

We have also considered three simple ad-hoc rules for evaluating the uncertainty due to finite resolution, including those recommended for the by the GUM and ISO 14253-2.

For the measurement process scenario and using the criterion that the (*k* = 2) expanded uncertainty should include approximately 95 % of the potential errors, we found that the ISO 14253-2 rule underestimates the uncertainty for roughly 30 % of the *μ*, *σ* values, while the GUM rule and Rule 3 do significantly better. Additionally, we pointed out that evaluating the standard deviation of repeated measurements using several different reference artifacts, with values that are not increments of the resolution unit, improves the containment probability for all the uncertainty rules examined.

For the special test scenario, where a large number of measurements are available to compute the mean value and the standard deviation, both the GUM and ISO 14253-2 rules greatly overestimate the uncertainty of the mean for large sample sizes. This overestimation results from setting a lower limit for the uncertainty equal to $\frac{1}{\sqrt{12}}$ regardless of sample size or the value of the standard deviation. Rule 3 more closely matches the expanded uncertainty to the 95th percentile error while still maintaining a 95 % error containment probability.

## Figures and Tables

**Table 1:** Measurement Process Scenario

	Rule 1 (GUM)		Rule 2 (ISO)		Rule 3		∞ Resolution
	*μ*_Ref_ = 0	*μ*_Ref_ = Σ*μ*	*μ*_Ref_ = 0	*μ*_Ref_ = Σ*μ*	*μ*_Ref_ = 0	*μ*_Ref_ = Σ*μ*	*U* = 2 *s*
Min P(*x* ≤ *U*)	0.75	0.84	0.65	0.74	0.85	0.91	0.94
Avg P(*x* ≤ *U*)	0.97	0.98	0.95	0.97	0.98	0.98	0.95
% P ≤ 0.94	13 %	7 %	30 %	19 %	10 %	5 %	0.00
Avg (*U* − |*ε*_95_|)	0.23	0.29	0.12	0.16	0.27	0.30	0.02
Max (*U* − |*ε*_95_|)	0.69	0.89	0.58	0.76	0.81	0.89	0.07
Min (*U* − |*ε*_95_|)	−0.13	−0.25	−0.38	−0.48	−0.15	−0.14	0.00

**Table 2:** Special Test Scenario

	Rule 1 (GUM)	Rule 2 (ISO)	Rule 3
Min P(*x* ≤ *U*)	1.0	1.0	0.94
Avg P(*x* ≤ *U*)	1.0	1.0	0.98
% P ≤ 0.94	0	0	1 %
Avg *U* − |*ε*_95_|	0.51	0.50	0.15
Max *U* − |*ε*_95_|	0.58	0.58	0.58
Min *U* − |*ε*_95_|	0.18	0.18	−0.01

## References

[1] International Standard (1998). Geometrical Product Specifications (GPS) - Inspection by measurement of workpieces and measuring instruments — Part 1: Decision rules for providing conformance or non-conformance with specification.

[2] International Organization for Standardization (1993). Guide to the Expression of Uncertainty in Measurement.

[3] International Standard (1999). Geometrical Product Specifications (GPS) - Inspection by measurement of workpieces and measuring instruments — Part 2: Guide to the Estimation of Uncertainty in GPS Measurement in Calibration of Measuring Equipment and in Product Verification.

[4] Stuart A, Ord JK (1998). Kendall’s Advanced Theory of Statistics: Distribution Theory.

[5] Elster C (2000). Evaluation of Measurement Uncertainty in the Presence of Combined Random and Analogue to Digital Conversion Errors. Measurement Science and Technology.

